# Assessment of carvacrol-antibiotic combinations’ antimicrobial activity against methicillin-resistant *Staphylococcus aureus*

**DOI:** 10.3389/fmicb.2023.1349550

**Published:** 2024-01-08

**Authors:** Deniz Al-Tawalbeh, Yazan Alkhawaldeh, Hana M. Sawan, Farah Al-Mamoori, Ali Al-Samydai, Amal Mayyas

**Affiliations:** ^1^Department of Medicinal Chemistry and Pharmacognosy, Faculty of Pharmacy, Yarmouk University, Irbid, Jordan; ^2^Aurum Biotech, Amman, Jordan; ^3^Department of Pharmaceutical Sciences, Faculty of Pharmacy, Zarqa University, Zarqa, Jordan; ^4^Pharmacological and Diagnostic Research Centre, Faculty of Pharmacy, Al-Ahliyya Amman University, Amman, Jordan; ^5^Faculty of Health Sciences, American University of Madaba, Madaba, Jordan

**Keywords:** carvacrol, MRSA, sulfamethoxazole, trimethoprim, linezolid, minocycline

## Abstract

**Introduction:**

This study aimed to assess the antimicrobial activity of carvacrol in combination with approved antibiotics against methicillin-resistant *Staphylococcus aureus* (MRSA). Carvacrol, a phenolic monoterpenoid component of essential oils, has demonstrated antimicrobial properties against gram positive and gram negative bacteria. The study evaluated the antimicrobial effects of carvacrol combined with sulfamethoxazole, linezolid, minocycline, and trimethoprim.

**Methods:**

The MRSA strain (ATCC-33591) was used, and various assays, including MIC determination, checkerboard assay, and microdilution assay were conducted.

**Results:**

The results showed that the combination of carvacrol with antibiotics yielded better outcomes compared to monotherapy, leading to reduced bacterial colonization. Carvacrol, sulfamethoxazole, and trimethoprim exhibited weak anti-staphylococcal effects, while linezolid and minocycline demonstrated stronger effects. This suggests that conventional antibiotic therapy may not be sufficient to effectively treat MRSA infections, potentially causing delays in healing or an exacerbation of the condition. Carvacrol combinations with two antibiotics displayed superior results compared to other pairs, indicating synergistic or additive effects of carvacrol with linezolid, minocycline, and sulfamethoxazole.

**Conclusion:**

These findings propose a new approach for developing drug molecules for MRSA treatment which combine volatile oils with available regimens. Further studies are recommended to evaluate the efficacy and biosafety of these combinations using *in vivo* or *ex vivo* models, aiming to minimize side effects and facilitate human trials. This study provides valuable insights into the potential use of carvacrol-antibiotic combinations as a novel therapeutic approach against MRSA.

## Introduction

Methicillin-resistant *Staphylococcus aureus* (MRSA) is a leading cause of nosocomial infection worldwide (Samaranayake et al., 2019). MRSA is frequently responsible for serious infectious disorders such as food poisoning, pyogenic endocarditis, supportive pneumonia, otitis media, osteomyelitis, pyogenic infections, soft tissues, and other ailments (Algammal et al., 2020). According to available statistics, MRSA is prevalent in Arab countries in the Middle East and North Africa (Borg et al., 2007). MRSA was found at the highest rates in hospitals in Jordan, Egypt, and Cyprus in the Mediterranean region. This could be due to hospital congestion, poor infection control practices and the uncontrolled use of antibiotics without a prescription, which has resulted in the emergence of communities of antibiotic-resistant organisms such as MRSA (Borg et al., 2007).

According to the guidelines, vancomycin, sulfamethoxazole, linezolid, minocycline, and trimethoprim are antibiotics recently used to treat MRSA-related infections (Moore et al., 2012). Vancomycin use is declining because of increasing resistance report and because the drug is failing to penetrate the tissue leading to slow bactericidal activity (Moore et al., 2012). Although linezolid inhibits protein binding by attaching to the 50S ribosomal subunit of the 23S rRNA site, MRSA encodes methyltransferase enzyme to prevent linezolid binding, resulting in resistance (Santiago et al., 2015). Trimethoprim and sulfamethoxazole are used in conjunction to treat MRSA infections; prior research has shown that when both antibiotics were coupled with other regimens, the outcomes improved (Cowan, 1999).

Because of antibiotic resistance, treating MRSA infections is tough. As a result, alternative remedies must be developed. Using plant-derived phytochemicals to boost the efficiency of conventional antibiotics is one of the fundamental approaches in alternative medicine (Santiago et al., 2015).

Plants produce four types of antimicrobial compounds: phenolics and polyphenols, terpenoids and essential oils, lectins and polypeptides, and alkaloids. Most bioactive plant extracts include complex combinations of these groups, and their combined activity can produce a greater effect (Cowan, 1999). Today, between 70% and 95% of people in many developing nations continue to rely on plants as their primary form of medication, and many governments have integrated traditional plant-based medicines into mainstream healthcare systems through regulations (Chassagne et al., 2021).

Essential oils represent a major group of plant antibacterial agents. Essential oils are complex volatile molecules spontaneously generated in various plant components during secondary metabolism. Essential oils have enormous potential in biomedicine since they successfully kill various bacterial, fungal, and viral diseases. Essential oils contain a variety of aldehydes, phenolics, terpenes, and other antimicrobial components that enhances their effectiveness against a wide range of diseases (Swamy et al., 2016). Such as in cinnamaldehyde; a broad spectrum anti-bacterial volatile oil that grants it’s activity from the available hydrogen bond, can exert its anti-bacterial activity by inhibiting the cell division (Li et al., 2015).

Aside from antibacterial activity, essential oils and their constituents can synergize with some antibiotics, boosting their antimicrobial activity. Carvacrol, a phenolic monoterpene isomeric with thymol, is a prominent component of the essential oils of Labiatae plants such as *Origanum* and *Thymus*. It has many reported biological activities such as anti-oxidant, anti-cancer, and antibacterial activities (Sharifi-Rad et al., 2018). Carvacrol, in particular, has been widely investigated in food as an antibacterial agent to control gram-positive and gram-negative microorganisms. Carvacrol has been shown to have synergistic effects when combined with a variety of antibiotics, including macrolides (Nostro and Papalia, 2012; Langeveld et al., 2014).

In this work, carvacrol’s antibacterial activity was tested against MRSA strain (ATCC-33591) alone and in combination with standard antibiotics (sulfamethoxazole, linezolid, minocycline, and trimethoprim) using agar well diffusion method, in which carvacrol was paired with either one or two antibiotics. In addition, a checkerboard assay was used to confirm the synergy of carvacrol-antibiotic combinations.

## Results and discussion

Microbial infections and antibiotic resistance are two of the most serious threats to society’s health today. Various approaches to combating antibiotic resistance have been proposed in recent years. Phytochemicals have demonstrated substantial antibacterial activity, and several studies have employed natural compounds to combat bacterial resistance. These compounds can be used alone or in conjunction with antibiotics to boost antibacterial activity against various bacteria (Khameneh et al., 2019).

Several earlier studies examined the antibacterial activities of essential oils and their key monoterpenes. Many terpenes are effective against a wide range of microorganisms, including gram-positive and gram-negative bacteria and fungi. Essential oils and their constituents’ antibacterial qualities are used in a wide range of commercial goods, including dental root canal sealers, antiseptics, food preservatives, and feed additives (Trombetta et al., 2005).

Carvacrol is a phenolic monoterpenoid component of essential oils found in many plants, e.g., oregano (*Origanum vulgare*), thyme (*Thymus vulgaris*), pepperwort (*Lepidium flavum*), wild bergamot (*Citrus aurantium bergamia*), and is reported to have antimicrobial, antioxidant, and anti-cancer activities (Sharifi-Rad et al., 2018). Many studies have demonstrated the potential antimicrobial activity of carvacrol against many Gram-positive and Gram-negative bacteria, including *Staphylococcus aureus, Staphylococcus epidermidis, Streptococcus pneumonia, Escherichia coli, Klebsiella pneumonia, Proteus mirabilis, Enterobacter* spp.*, Serratia* spp. (Bnyan et al., 2014) *Bacillus cereus* (Ultee and Smid, 2001), *Listeria monocytogenes* (Karatzas et al., 2001), and *Pseudomonas aeruginosa* (Ghizlane et al., 2011), as well as fungi; e.g., *Aspergillus niger, Aspergillus fumigatus, Aspergillus flavus, Aspergillus ochraceus, Alternaria alternata, Botrytis cinerea, Cladosporium* spp.*, Penicillium citrinum, Penicillium chrysogenum, Fusarium oxysporum, Rhizopus oryzae* (Abbaszadeh et al., 2014) and *Cryptococcus neoformans* (Nobrega et al., 2016).

**Figure 1:**
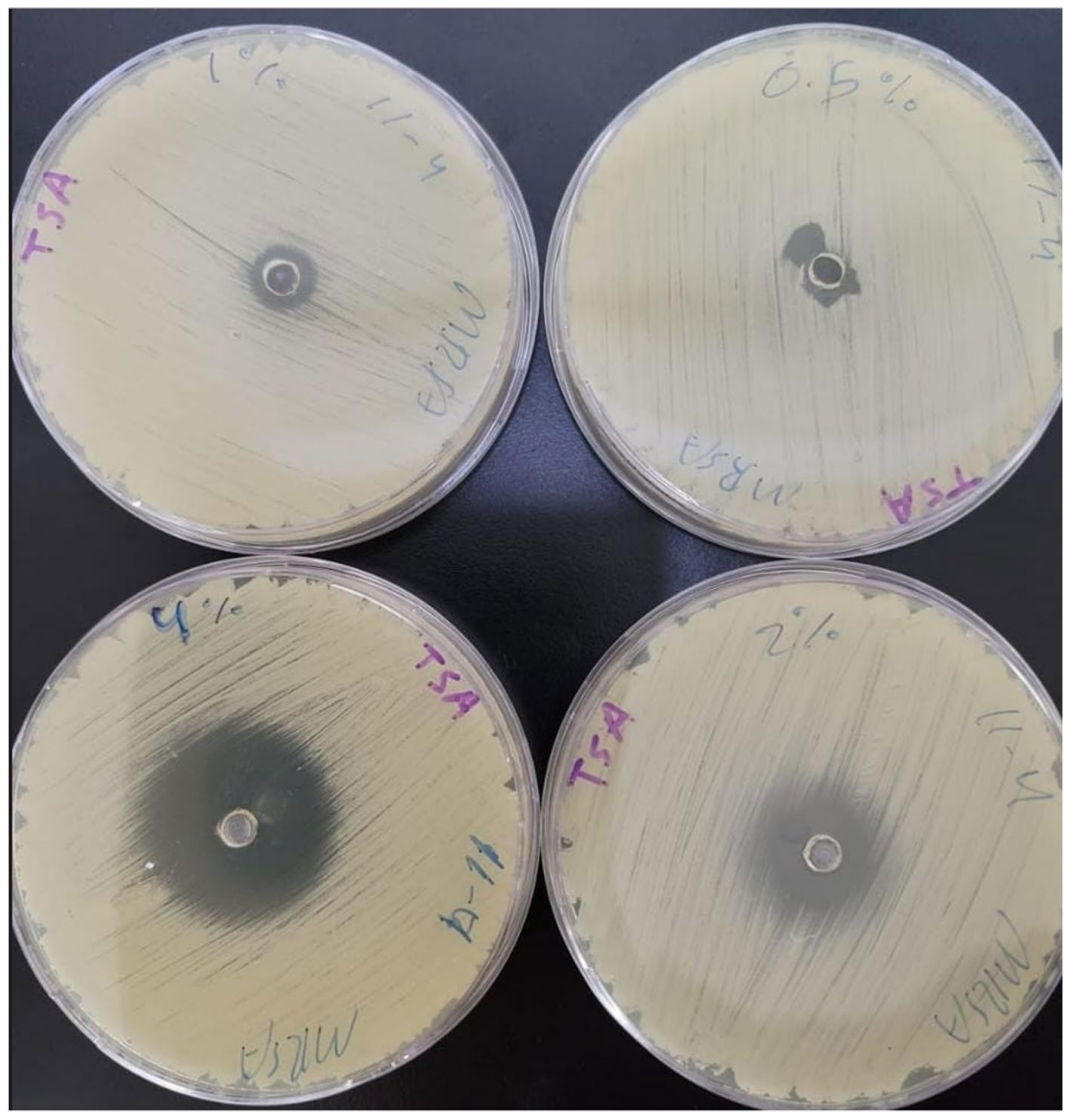
The inhibition zones of carvacrol with different concentrations (400, 200, 100, and 0.5 mg/mL).

In the current study, carvacrol was tested against MRSA (ATCC-33591), alone and in combination with conventional antibiotics (i.e., sulfamethoxazole, linezolid, minocycline, and trimethoprim). When tested alone using an agar diffusion assay, only minocycline yielded a 25 mm zone of inhibition, while carvacrol 400 mg/mL yielded a 35 mm zone of inhibition (Figure 1); thus, empirical therapy may not cover the treatment, leading to some delay in infection healing or worsening of the condition. Regarding MIC values, carvacrol, sulfamethoxazole, and trimethoprim all exhibited weak anti-staphylococcal effect with a MIC value of 4,000 μg/mL. In comparison, linezolid and minocycline exhibited good anti-staphylococcal effect with MIC values of 250 μg/mL (Table 1).

**Table 1:** Zone of inhibition and MIC values for each tested product alone.

Tested product	Structure	Zone of inhibition (mm)	MIC (μg/mL)	IC_50_ (mg/mL)
Carvacrol 400 mg/mL	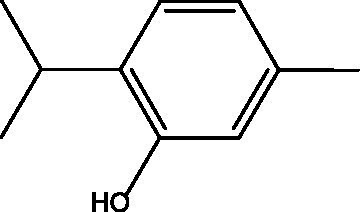	35	4,000	0.3689
Carvacrol 200 mg/mL	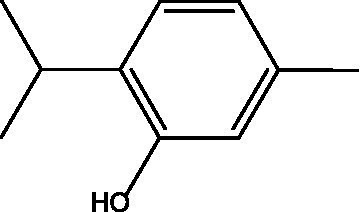	25	—	—
Carvacrol 100 mg/mL	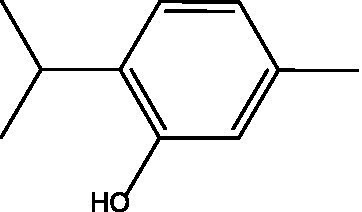	12	—	—
Carvacrol 0.5 mg/mL	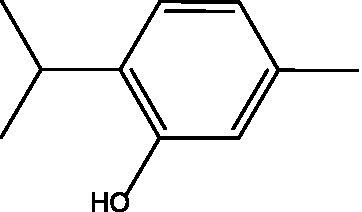	10	—	—
Carvacrol 0.2 mg/mL	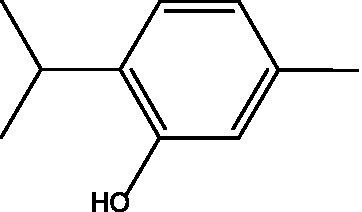	NA	—	—
Linezolid	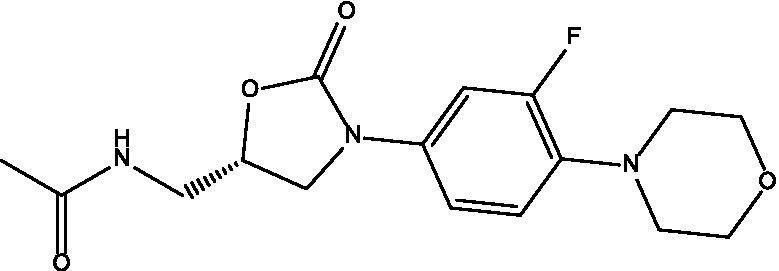	NA	250	0.0334
Minocycline	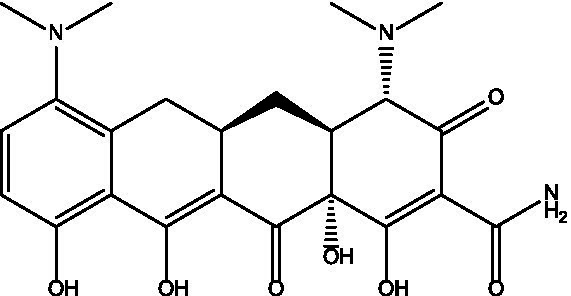	25	250	0.4471
Sulfamethoxazole	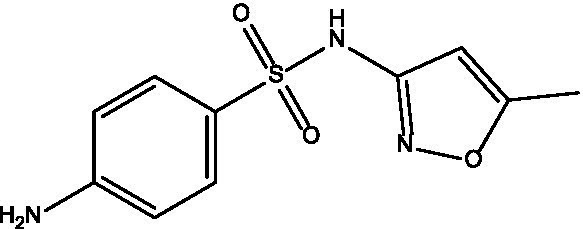	NA	4,000	0.6536
Trimethoprim	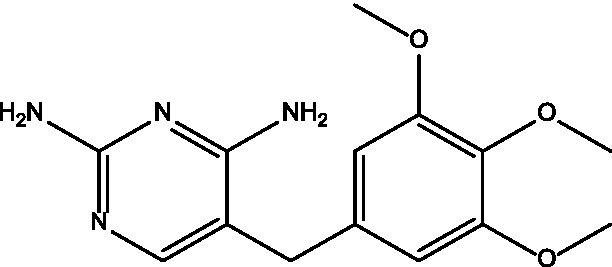	NA	4,000	0.0637

NA, no appearance for inhibition zone; full zone, no growth at all on the plate.

The anti-staphylococcal properties of carvacrol vary greatly in the literature (with MIC values ranging from 150 to 3,810 g/mL; Karatzas et al., 2001; Ghizlane et al., 2011). This variance can be attributable to various factors, including the strain utilized, the technique used, and even the solvent used to dissolve carvacrol, with ethanol having considerably lower MIC values than DMSO (Abbaszadeh et al., 2014).

Similarly, a study has also found a weak anti-staphylococcal effect of carvacrol against MRSA (ATCC 33591) using disk volatilization tests up to a concentration 1,000 μg/disc (Wang et al., 2016). However, another recent study reported a good anti-staphylococcal effect against the same strain (i.e., MRSA ATCC-33591), with a MIC value of 150 μg/mL (Selvaraj et al., 2020).

**Figure 2:**
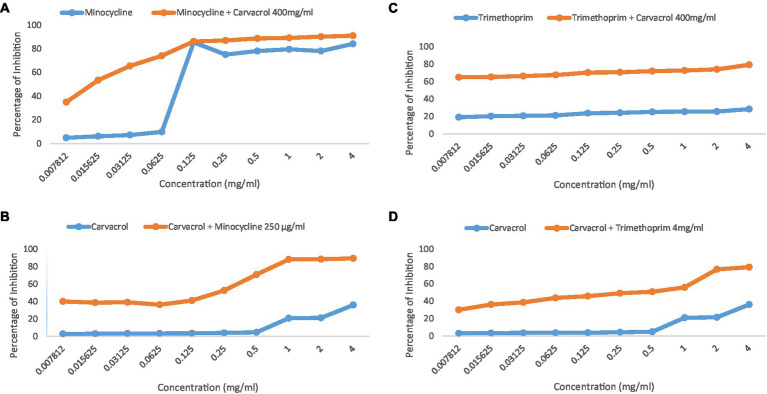
The synergistic effect achieved from carvacrol-antimicrobial combination. **(A)** Carvacrol 400 mg/mL with minocycline serially diluted. **(B)** Minocycline 250 μg/mL with carvacrol serially diluted. **(C)** Carvacrol 400 mg/mL with trimethoprim serially diluted. **(D)** Trimethoprim 4 mg/mL with carvacrol serially diluted.

On the other hand, when carvacrol was tested in combination with sulfamethoxazole, linezolid, minocycline, and trimethoprim, all combinations yielded full zones of inhibition (i.e., no growth at all on the plate; Table 2). In addition, when carvacrol was tested with two ‘antibiotics’ combinations, carvacrol combinations were superior to all tested pairs of antibiotics, where carvacrol combinations yielded either full zones of inhibition compared to no zone of inhibition (i.e., sulfamethoxazole + linezolid, sulfamethoxazole + trimethoprim, linezolid + minocycline, linezolid + trimethoprim), or a slightly improved zone of inhibition compared with the non-carvacrol combination (i.e., sulfamethoxazole + minocycline and minocycline + trimethoprim; Tables 2, 3). Furthermore, when a checkerboard assay was used to confirm the synergy of carvacrol-antibiotic combinations, FIC values suggested either an additive effect (i.e., carvacrol + linezolid) or synergistic effect (i.e., carvacrol + minocycline and carvacrol + trimethoprim; Tables 3, 4; Figure 2).

**Table 2:** Zone of inhibition for combined antibiotics.

Tested combination	Zone of inhibition (mm)	Tested combination	Zone of inhibition (mm)
Minocycline + sulfamethoxazole	21	Minocycline + carvacrol	Full zone
Minocycline + trimethoprim	22	Linezolid + carvacrol	Full zone
Minocycline + linezolid	NA	Sulfamethoxazole + carvacrol	Full zone
Linezolid + sulfamethoxazole	NA	Trimethoprim + carvacrol	Full zone
Linezolid + trimethoprim	NA	DMSO	NA
Sulfamethoxazole + trimethoprim	NA		

NA, no appearance for inhibition zone; full zone, no growth at all on the plate.

**Table 3:** Zone of inhibition for two antibiotics combined with carvacrol.

Tested combination	Zone of inhibition (mm)	Interpretation
Carvacrol + sulfamethoxazole + linezolid	22	NZI
Carvacrol + sulfamethoxazole + minocycline	23	Improved
Carvacrol + sulfamethoxazole + trimethoprim	18	NZI
Carvacrol + linezolid + minocycline	22	NZI
Carvacrol + linezolid + trimethoprim	14	NZI
Carvacrol + minocycline + trimethoprim	28	Improved

NZI, new zone of inhibition occurrence when compared with two antibiotics combination alone; Improved, the new zone of inhibition is larger than the zone of inhibition achieved by combining two antibiotics.

In spite of its promising antimicrobial activity, few studies have investigated the synergistic activity of carvacrol with some conventional antibiotics. For example, in a study conducted to evaluate the carvacrol-erythromycin synergistic effect against erythromycin-resistant Group A Streptococci, highly significant synergy (FIC Index ≤ 0.5, *p* < 0.01) was detected in 21 out of 32 strains (Magi et al., 2015). In another study, carvacrol showed synergy in combination with oxacillin against 7 out of 10 MRSA clinical strains and membrane-damaging capacity against one of the tested strains (Köse and Özyurt, 2023).

**Table 4:** FIC values for the combinations with carvacrol.

Tested product	FIC	Interpretation	MIC before combination (μg/mL)	MIC after combination (μg/mL)
Carvacrol + minocycline	0.046	Synergistic effect	Carvacrol = 4,000	Carvacrol = 62.5
			Minocycline = 250	Minocycline = 7.8
Carvacrol + linezolid	1.004	Additive effect	Carvacrol = 4,000	Carvacrol = 15.6
			Linezolid = 250	Linezolid =250
Carvacrol + sulfamethoxazole	1.125	Indifferent effect	Carvacrol = 4,000	Carvacrol = 500
			Sulfamethoxazole = 4,000	Sulfamethoxazole = 4,000
Carvacrol + trimethoprim	0.039	Synergistic effect	Carvacrol = 4,000	Carvacrol = 31.25
			Trimethoprim = 4,000	Trimethoprim = 125

The FIC index is interpreted as <0.5 is a synergistic effect, 0.5–1 is an additive effect, 1–4 is an indifferent effect, and more than 4 is an antagonistic effect.

A previous study that compared different volatile oils, including carvacrol, against gram-positive and gram-negative bacteria demonstrated that the specific structure features of carvacrol, which are the hydrophobicity and the presence of the free hydroxyl group, may attribute to the antimicrobial activity and enhance its bioavailability (Nostro and Papalia, 2012). Regarding the mechanism of action, Abdelmounaïm et al. suggested that carvacrol destroys bacterial cell walls of MRSA (ATCC 43300) in a concentration-dependent manner through reduction of ATP synthesis and membrane electrical potential, which ultimately enhances membrane permeability to other antibiotics (Abdelmounaïm et al., 2013). Other possible explanations for its antibacterial activity include hydroxyl group and a delocalized electron system in its structure (Marinelli et al., 2019). In a study that was conducted by Soumya et al., revealed that the hydrophobicity and the hydroxyl group are responsible for the interference in the biofilm formation by diffusing through the polar polysaccharide matrix thus disturbing the membrane and inhibiting the biofilm formation (Ghizlane et al., 2011). Another study in 2019 approved that carvacrol disrupted the membrane integrity of both *S. aureus* (ATCC 25923) and MRSA clinical isolates by acting as efflux pump inhibitors and down-regulate the mepA gene of efflux pump in MRSA (Mouwakeh et al., 2019).

In addition, another recent study demonstrated that despite the nonfatal effect of carvacrol against MRSA (ATCC-33591), carvacrol exhibited anti-biofilm efficacy through the reduction of biofilm-associated slime and extracellular polysaccharide production (Selvaraj et al., 2020). The same study reported the anti-virulence effect of carvacrol against MRSA (ATCC-33591) through inhibition of the antioxidant pigment staphyloxanthin. Furthermore, another study reported several putative mechanisms for the cell damage of *S. aureus* caused by oregano essential oil (carvacrol-rich oil), including changes in the integrity of the cell membrane and cell morphology, as well as disturbing protein synthesis and amino acid metabolism (Hao et al., 2021).

Another interesting approach for tackling the mechanism of action of antibacterial compounds is to study bacteria at the single-cell level unveiling the morphological, physiological and genetic changes in bacteria when exposed to inhibitory substances in ways not attainable by studying large populations using traditional culturing methods. A study conducted by Kong et al. adopted this approach to record morphological changes in *Escherichia coli* during ampicillin exposure and to quantify the minimum inhibitory concentration of the antibiotic using a simple low-cost agarose membrane resting on a double-sided adhesive tape (Kong et al., 2019).

The results of this study pave the way for testing other carvacrol-conventional ‘antibiotics’ combinations, possibly using high throughput automated platforms, which enable large-scale screens for antibacterial compounds that use robotic instrumentation to dispense a precise amount of MRSA putative antimicrobials, followed by automated microscopy and image analysis (Rajamuthiah et al., 2014). One such promising assay is the multistep modular-based microfluidic system, which can perform MRSA identification and provide information on drug susceptibility simultaneously (Chen et al., 2013). However, more extensive safety examinations and *in vivo* tests are required before carvacrol can be employed in the future.

## Materials and methods

### Phytocompound and antibiotics

Carvacrol was obtained from Sigma-Aldrich (United States) and prepared as 400 mg/mL (w/v) in dimethyl sulfoxide (DMSO; Fisher Scientific, United Kingdom) and phosphate buffer saline (PBS; Pan Biotic, Germany; 1:1) as solution stock. Minocycline, linezolid, trimethoprim, and sulfamethoxazole laboratory standards were obtained from the Al-Hikma pharmaceutical industry and freshly dissolved in DMSO/PBS (1:1) to obtain a 12 mg/mL concentration. Stock solutions were used later on to prepare further dilutions in DMSO:PBS (1,1) solvent.

### Bacterial strain and growth conditions

The MRSA strain (ATCC-33591) was used in this study. Bacterial strains were inoculated on tryptone soya agar (TSA; Biolab, Hungary), and incubated at 37°C for 24 h. Subcultures were kept in trypticasein soy broth (TSB; Condalab, Spain) supplemented with 15% glycerol and stored at −80°C for later use.

### Antimicrobial sensitivity testing and minimum inhibitory concentration assay

The susceptibility of carvacrol and antibiotics was tested using the well diffusion method on solid tryptone soya agar. Plates were incubated at 37°C for 24 h.

Clinical and Laboratory Standards Institute (CLSI, 2020) recommendations were followed to determine the MIC using the broth dilution method in 96-well microplates. DMSO:PBS (1:1) was used as negative control. Cultures of (0.5 × 10^8^) MRSA were used and incubated with carvacrol and each antibiotic for 24 h at 37°C, well’s final concentration was in the range (4,000–7.81 μg/mL). After incubation, the MIC values were determined visually, and the optical density (OD) at 600 nm was measured using the microplate reader spectrophotometer (Mutiskan Go Thermo Scientific, United States).

### Checkerboard assay

The effect of combining carvacrol with the antibiotics and combining different antibiotics was determined using a two-fold broth Microdilution assay. Carvacrol and antibiotics were dissolved in DMSO: PBS in a ratio of 1:1 and serially diluted. The 96-well microplate was used with a final volume of 200 μL in each well. At the end of the Microdilution, the concentration range was (4,000–7.81 μL/mL). An inoculum concentration of (0.5 × 108) was used using freshly grown bacteria. The plate was incubated for 24 h at 37°C. DMSO:PBS (1:1) was the negative control.

The MICs were measured using the microplate reader spectrophotometer, and the fractional inhibitory concentration (FIC) was calculated for each combination using the formula:


$$\begin{array}{l} \mathrm{FIC}=\left(\begin{array}{l} \mathrm{MIC}\;\mathrm{of compound}\;1\;\mathrm{in combination}\\ /\mathrm{MIC}\;\mathrm{of compound}\;1\;\mathrm{alone} \end{array}\right)\\ \;\;\;\;\;\;\;\;\;\;\;\;\;\;+\left(\begin{array}{l} \mathrm{MIC}\;\mathrm{of compound}\;2\;\mathrm{in combination}\\ /\mathrm{MIC}\;\mathrm{of compound}\;2\;\mathrm{alone} \end{array}\right) \end{array}$$

The FIC index is interpreted as; <0.5 is a synergistic effect, 0.5–1 is an additive effect, 1–4 is an indifferent effect, and more than 4 is an antagonistic effect.

The percentage of inhibition was calculated using the following formula:


$$\mathrm{Percentage of inhibition}=\left(\begin{array}{l} \mathrm{control}\;\mathrm{OD}-\\ \left(\begin{array}{l} \mathrm{sample}\;\mathrm{OD}\\ /\mathrm{control}\;\mathrm{OD} \end{array}\right) \end{array}\right)\times 100\%$$

## Conclusion

To the best of our knowledge, this is the first study that describes the potential effect of carvacrol when combined with one or two approved antibiotics. The combination of carvacrol with antibiotics gives better results than monotherapy. The overall reduction in bacterial colonization gives a new approach to developing new drug molecules against MRSA treatment. The efficacy and biosafety of the proposed combinations may be studied later on using *in vivo* or *ex vivo* designs in order to minimize the side effects or difficulties in human trials.

## Data availability statement

The original contributions presented in the study are included in the article/supplementary material, further inquiries can be directed to the corresponding author.

## Author contributions

DA: Conceptualization, Investigation, Methodology, Supervision, Visualization, Writing – original draft. YA: Methodology, Writing – original draft. HS: Methodology, Validation, Writing – original draft. FA: Conceptualization, Data curation, Resources, Writing – review & editing. AA: Formal analysis, Software, Writing – review & editing. AM: Methodology, Validation, Visualization, Writing – review & editing.
